# Early changes in emotional processing as a marker of clinical response to SSRI treatment in depression

**DOI:** 10.1038/tp.2016.130

**Published:** 2016-11-22

**Authors:** B R Godlewska, M Browning, R Norbury, P J Cowen, C J Harmer

**Affiliations:** 1 Psychopharmacology Research Unit, University Department of Psychiatry, University of Oxford, Oxford, UK; 2 Psychopharmacology and Emotion Research Laboratory, University Department of Psychiatry, Warneford Hospital, University of Oxford, Oxford, UK; 3 Department of Psychology, Whitelands College, University of Roehampton, London, UK

## Abstract

Antidepressant treatment reduces behavioural and neural markers of negative emotional bias early in treatment and has been proposed as a mechanism of antidepressant drug action. Here, we provide a critical test of this hypothesis by assessing whether neural markers of early emotional processing changes predict later clinical response in depression. Thirty-five unmedicated patients with major depression took the selective serotonin re-uptake inhibitor (SSRI), escitalopram (10 mg), over 6 weeks, and were classified as responders (22 patients) versus non-responders (13 patients), based on at least a 50% reduction in symptoms by the end of treatment. The neural response to fearful and happy emotional facial expressions was assessed before and after 7 days of treatment using functional magnetic resonance imaging. Changes in the neural response to these facial cues after 7 days of escitalopram were compared in patients as a function of later clinical response. A sample of healthy controls was also assessed. At baseline, depressed patients showed greater activation to fear versus happy faces than controls in the insula and dorsal anterior cingulate. Depressed patients who went on to respond to the SSRI had a greater reduction in neural activity to fearful versus happy facial expressions after just 7 days of escitalopram across a network of regions including the anterior cingulate, insula, amygdala and thalamus. Mediation analysis confirmed that the direct effect of neural change on symptom response was not mediated by initial changes in depressive symptoms. These results support the hypothesis that early changes in emotional processing with antidepressant treatment are the basis of later clinical improvement. As such, early correction of negative bias may be a key mechanism of antidepressant drug action and a potentially useful predictor of therapeutic response.

## Introduction

Major depression is associated with a range of negative biases in the processing of emotional information.^1^ For example, compared with healthy controls, depressed patients selectively recall more negative, self-related emotional information in memory tasks and demonstrate negative biases in the perception of social signals such as emotional facial expressions.^2, 3^ Such biases have been associated with aberrant responses across a network of neural areas involved in emotional processing. Untreated patients with depression show enhanced reactivity to negative stimuli in networks involved in emotional salience and attention such as the amygdala, visual cortex, insula and thalamus as well as differences in response in areas thought to have a role in emotion monitoring, evaluation and regulation including the anterior cingulate (ACC) and dorsolateral prefrontal cortex.^4, 5, 6, 7^ These negative biases appear to have a key role in the pathophysiology and maintenance of depressive states.^1^

We have proposed that the therapeutic effect of antidepressant drugs is mediated by early reversal of these negative emotional biases.^8^ For example, in healthy volunteers, 7 days' treatment with the selective serotonin re-uptake inhibitor (SSRI), citalopram, and the selective noradrenaline re-uptake inhibitor, reboxetine, diminished the recognition of negative emotional faces, increased recall of positive self-referential words and attenuated the amygdala response to fearful faces as measured by functional magnetic resonance imaging (fMRI).^9, 10, 11^ Studies in depressed patients have also shown attenuation of the neural response to sad and fearful faces after antidepressant treatment in limbic areas including the amygdala, insula and ACC.^4, 7, 12^ However, these studies have typically been conducted after 6–8 weeks of therapy, by which time clinical response is well established. As such, it is difficult to assess whether changes in neural responsivity are a cause or effect of changes in depression symptoms.

To address this question, we recently assessed the effects of just 7 days' treatment with the SSRI, escitalopram (10 mg), in depressed patients using a double-blind, placebo-controlled study.^13^ Amygdala response to fearful versus happy faces was normalised following escitalopram relative to placebo treatment before any significant clinical response. Such early changes in processing may have a critical role in the emergence of clinical therapeutic effects over time as the patient responds to the reduced impact of negative events, stressors and cues.^8^ Early changes in information processing were seen in both behavioural and fMRI studies, and may lead to a gradual change in clinical symptomatology through interaction with on-going environmental and social stimuli.

If this hypothesis of antidepressant action is correct, we would expect early changes in emotional processing to be predictive of later clinical response to SSRI therapy in depression. The current study tested this critical hypothesis using a functional neuroimaging emotional paradigm. We predicted that a greater reduction in the neural response to fearful versus happy facial expressions across a network of previously identified brain areas, including the amygdala, insula and cingulate cortex, after 1 week of treatment would be associated with an increased response to treatment at week 6. We did not use placebo treatment because of ethical concerns about giving placebo for 6 weeks to depressed patients. In addition, our above-mentioned study^13^ showed that after 7 days of treatment with escitalopram the amygdala activity in depressed patients was similar to that in healthy controls, whereas placebo produced no such effect. Healthy controls were included to show baseline differences between the tested groups, and to explore whether there were differences between controls and responders and non-responders.

## Materials and methods

### Participants

Thirty-five participants (20 F:15M) with major depression completed the fMRI and treatment protocol (see Table 1). An additional four patients consented to take part, but dropped out before the end of the 6-week period of treatment (two after the first session and two before the 6-week assessment). Thirty-one healthy control participants completed baseline scans; however, data from two participants were lost because of computer error, leaving a sample of 29 (17 F:12M). All participants were assessed for the presence of current and past psychiatric disorder with the Structured Clinical Interview for DSM-IV^14^. The depressed patients met the criteria for a primary diagnosis of major depressive disorder; the control participants had no current or lifetime diagnosis. Exclusion criteria for the study included psychosis or substance dependence as defined by DSM-IV, being at clinically significant risk of suicidal behaviour, having contraindications to escitalopram treatment or being treated with psychotropic medication less than 3 weeks before the study (5 weeks for fluoxetine). We also excluded patients with major somatic or neurological disorders, who were pregnant or breast-feeding, with any contraindications to MRI or concurrent medication, which could alter emotional processing. All participants were right-handed. The study was approved by the Oxford Research Ethics Committee, and all participants gave written informed consent.

### Study design and drug treatment

The sample of patients (*n*=35) and healthy controls (*n*=29) were assessed at baseline. Patients received 10 mg escitalopram each morning for a period of 6 weeks without dose adjustment. In the patient group, assessment of depressive severity and treatment response was made using the Hamilton Depression Rating Scale (HAM-D),^15^ Beck Depression Inventory (BDI)^16^ and Spielberger's State-Trait Anxiety inventory (STAI)^17^ at baseline, week 1 and week 6. The fMRI assessments were completed at the same time points. The current analysis focuses on the degree to which early changes (between baseline and week 1) in the function of emotional processing systems was able to predict clinical response at week 6 in the depressed patients. After the 6-week duration of the study, all patients were offered treatment openly with escitalopram according to usual clinical practice (data not captured). Clinical response to the SSRI was defined as a reduction in HAM-D of 50% or more from baseline after 6 weeks of treatment.^18^ Healthy controls were assessed at baseline only with HAM-D, BDI and STAI, and had a single fMRI scan performed at the same time.

### fMRI data acquisition

fMRI data were acquired on a 3 T Siemens TIM TRIO (Siemens, Erlangen, Germany). Data were acquired with a voxel resolution of 3 × 3 × 3.5 mm, repetition time (TR)/ echo time (TE)/ flip angle=2000 ms/28ms/89^o^. A total of 256 volumes were acquired in an experiment lasting 8.5 min. T_1_-weighted structural images were acquired using a magnetisation-prepared rapid acquisition by gradient echo sequence with a voxel resolution 1.0 × 1.0 × 1.0 mm on a 208 × 256 × 200 grid, TE/ inversion time/TR=4.68/900/2040 ms. To monitor cardiac and respiratory processes, subjects wore a pulse oximeter and respiratory bellows.

### fMRI experimental task

During fMRI scanning, participants completed a well-validated gender discrimination task involving the rapid presentation of fearful and happy faces.^12^ This task, which involves passive processing of emotional information, under the form of fearful and happy faces, with minimal attentional effort, provides an incidental measure of emotional processing and is believed to be a better probe of limbic function.^19^ In this task, nine 30-s blocks of a baseline fixation cross were interleaved with eight 30-s blocks of the emotional task (four blocks of fear and four blocks of happy). Each face was presented for 100 ms, and the subjects were asked to report the gender of the face via an MRI-compatible key pad.

### fMRI preprocessing and statistical analysis

fMRI data were preprocessed and analysed using FMRIB Software Library (FSL).^20^ Briefly, motion correction was applied using a rigid body registration to the central volume; the brain matter was segmented from non-brain using a mesh deformation approach. Gaussian spatial smoothing was applied with a full-width half-maximum of 5 mm; high-pass temporal filtering was applied using a Gaussian-weighted running line filter, with a 3 dB cutoff of 120 s.

A general linear model was fitted in pre-whitened data space. Two explanatory variables (plus their temporal derivatives) were modelled: ‘fear faces' and ‘happy faces'. All explanatory variables were convolved with a default haemodynamic response function (Gamma function, delay=6 s, s.d.=3 s), and filtered by the same high-pass filter as the data. The impact of physiological noise on the BOLD signal was reduced using the Physiological Noise Modelling tool of FSL. Pulse oximetry and respiratory bellows' data were processed by Physiological Noise Modelling to create 33 nuisance regressors that were added to the first-level fMRI model. The full model was simultaneously regressed against the BOLD data, giving the best-fitting amplitudes for each explanatory variable while accounting for the physiological noise.

The task contrast of interest in this study was the relative activation of fearful versus happy faces. The degree to which the change in neural activity in this contrast, induced by 1 week of SSRI treatment, predicted participants' clinical response on the HAM-D to medication over 6 weeks was tested using a three-level analysis. The first level consisted of the fearful versus happy contrast maps, as described above, calculated for each depressed subject and each visit. Second-level, fixed effects analyses calculated the degree to which these difference maps changed across the first week of treatment for each depressed subject. Lastly, a third-level, between-subject, random effects analysis assessed whether this change in neural activity differed between depressed patients who went on to respond to the medication and those who did not. Baseline HAM-D score was included as a regressor in the third-level analysis to account for the potential influence of initial depression severity effects on both early neural differences and overall clinical response (NB although baseline HAM-D score did not differ between the two groups, responders and non-responders (Table 1) and equivalent results were obtained when the analysis was run without this covariate).

The results of all group-level analyses were corrected across the whole brain using cluster-based thresholding with a height threshold of *Z*>2.3 and a (whole-brain-corrected) spatial extent threshold of *P*=0.05. We also defined bilateral amygdala, bilateral insula and ACC cortex masks as *a priori* regions of interest based on their sensitivity to early effects of antidepressant drug treatments in previous studies.^21^ These masks were derived from the Harvard–Oxford Cortical and Subcortical anatomical atlases and were used in small volume correction analyses (clusters determined by *Z*>2.3 and a (corrected) cluster significance threshold of *P*=0.05) of the data. All reported analyses are corrected across the whole brain unless otherwise specified (labelled as small volume correction (SVC)). Lastly, the causal relationship between early change in fMRI signal, change in symptom score across the first week of treatment and response status following 6 weeks of treatment was examined using mediation analyses. The mean change in activity within the functional clusters identified in the previous, predictive analyses was entered as independent variables in separate mediation analyses. In these analyses, patient response status was entered as the dependent variable and the change in symptom score across the first week was entered as a mediating variable. These analyses therefore tested whether the change in fMRI signal directly predicted future response to the antidepressant, or whether the neural effect on treatment response was significantly mediated by the initial change in symptoms—which would suggest that the fMRI signal acted simply as a marker of early symptom change. A bootstrapping procedure, as implemented by the PROCESS command for SPSS with 10 000 samples, was used to determine the confidence intervals of the direct and indirect effects of the mediation models.

## Results

### Clinical and demographic data

After 6 weeks' escitalopram treatment, 22 out of 35 patients (62%) were classified as responders. There were no differences between responders and non-responders in terms of gender, age, baseline depression severity, baseline trait anxiety or duration of current episode (See Table 1, all effect sizes <0.45 Cohen's *d*). However, week 6 responders versus non-responders showed numerically greater improvements in HAM-D score after 7 days of treatment, although this did not reach statistical significance (*t*=1.8, degree of freedom=32.3, *P*=0.08, Cohen's *d'* effect size 0.57).

#### fMRI data

**Figure 1:**
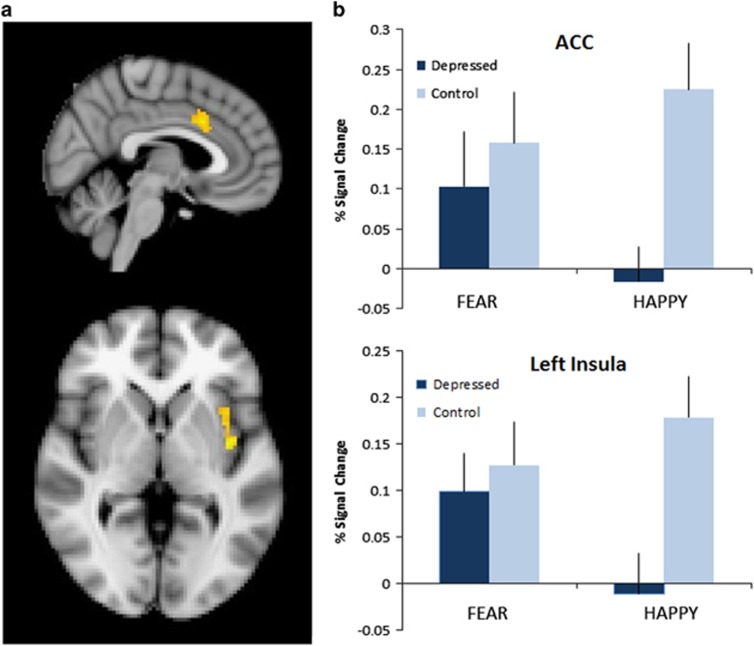
Baseline differences in the processing of negative versus positive affective stimuli differ between depressed patients and non-depressed control subjects. (**a**) Results of SVC-corrected analyses in the anterior cingulate and left insula; (**b**) extracted signal change from the identified clusters. All analyses were thresholded at *z*=2.3 and cluster-corrected with a FWE *P*<0.05. ACC, anterior cingulate cortex; FWE, family wise error; SVC, small volume correction.

At baseline, across all participants, fearful compared with happy faces activated a network of areas previously implicated in threat-relevant face processing. This included bilateral amygdala, bilateral insula and left middle temporal gyrus (Supplementary Table 1). Depressed patients displayed significantly greater pretreatment activation than controls to fearful versus happy faces in both the left insula (*P*=0.02, family wise error (FWE)-corrected, SVC) and ACC cortex (*P*=0.02, FWE-corrected, SVC; see Figure 1).

**Figure 2:**
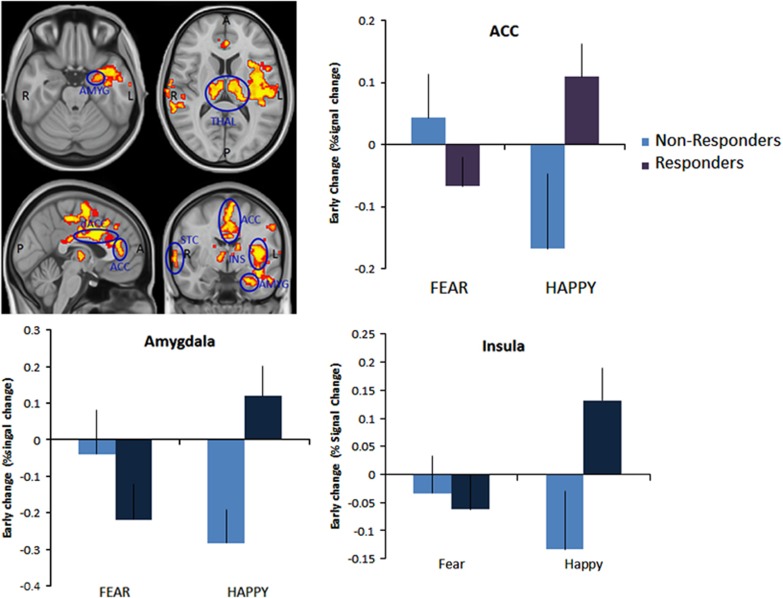
Early changes in the processing of negative versus positive affective stimuli predict week 6 treatment response to escitalopram across a network of areas involved in emotional processing. ACC, anterior cingulate cortex; AMG, amygdala; INS, insula; STC, superior temporal cortex; THAL, thalamus. Whole-brain-corrected analysis at *z*=2.3, responders versus non-responders.

Early changes in fMRI response to fearful versus happy faces following 7 days' escitalopram were predictive of later clinical response, controlling for baseline depression HAM-D severity. Week 6 responders showed a greater decrease in neural response to fearful versus happy faces following 7 days' escitalopram in the left amygdala, insula, anterior and posterior cingulate, bilateral supramarginal gyri and bilateral thalamus (*P*<0.05, FWE-corrected; see Table 2 and Figure 2). These clusters anatomically overlapped with the left insula and ACC clusters identified in the baseline comparison between depressed and control participants described above. A formal conjunction analysis identified significant clusters within both regions^22^ (ACC cluster: max *Z*-value=3.1, corrected *P*-value=0.05, coordinates=45, 70, 53; insula cluster: max *Z*-value=3.1, corrected *P*-value=0.005, coordinates=64, 61, 36).

A number of additional covariates were entered into the model to check the specificity of this effect. In this control analysis, early change in HAM-D after 1 week, baseline trait anxiety and week 6 change in trait anxiety were entered in as regressors of no interest. The results from this whole-brain analysis confirmed that early change in neural response to faces of fear versus happiness predicted clinical response even after accounting for these possible confounds (Supplementary Table 2).

It is possible that baseline differences in neural markers of emotional processing between responders and non-responders might also correlate with clinical outcome at 6 weeks, and such baseline effects could also contribute to the relationship between change in emotional processing over 7 days of treatment and later therapeutic response. However, we found no statistically significant differences to fearful versus happy faces at baseline (before treatment) in responders versus non-responders at a whole brain level or within the regions of interest. Interestingly, analysis of the fMRI data after 1 week of treatment alone was sufficient to predict outcome at 6 weeks with the same network of regions being involved (insula, thalamus, amygdala *svc*, thalamus and cingulate, see Supplementary Table 3). These additional analyses suggest that it is the ability of escitalopram to modify emotional processing early in treatment rather than baseline reactivity, which is related to later individual clinical response.

Lastly, we employed mediation analyses to test whether the relationship between early change in fMRI measures and response to treatment at week 6 was mediated by the change in depressive symptoms across the first week—that is, whether the fMRI measures reflected early symptom change rather than being directly related to later response. Separate mediation analyses were performed for each of the functional clusters identified in Table 2. In all of these analyses, the direct effect of the fMRI signal on response status remained significant (all *P*<0.012) after controlling for the mediating effects of early symptom change. In none of the analyses was the indirect effect (via early symptom change) significant (all bootstrapped confidence intervals spanned 0). In other words, mediation analysis provided additional evidence that the observed change in fMRI signal was directly related to the response to antidepressants and was not merely a marker of initial symptom response.

## Discussion

The principal finding of our study is that changes in neural processing of emotional information in the first week of SSRI treatment in depressed patients are predictive of short-term (6 weeks) therapeutic response. This early change in neural activity preceded clinically significant changes in depressive symptomatology and was not dependent on concurrent change in symptoms at the time of the second scan. The changes in neural activity occurred in regions which, at baseline, were differentially activated by fearful versus happy faces and which showed hyperactivity in depressed relative to control participants. As such, early normalisation of the neural systems responsible for negative processing biases in depression may be an important mechanism of antidepressant action.

The results are consistent with the cognitive neuropsychological theory of antidepressant action, which proposes that conventional antidepressant medications act through an early remediation of the negative emotional biases that characterise the depressed state.^8^ This theory hypothesises that treatments for depression reduce the overwhelming influx of automatic negative cues early in treatment, before the patient being aware of any changes in subjective state or in the clinical symptoms of depression. This early change in emotional processing, however, is the mechanism that drives the later clinical response as the patient's day-to-day environment is re-experienced through this more positive emotional perspective.^8^

Neural networks involving the amygdala, insula, cingulate and thalamus have been implicated in the generation of negative emotional biases in depression,^2, 21^ and our baseline comparison between the healthy controls and depressed patients replicated the pattern of increased response to negative versus positive faces in parts of this circuit. Previous studies have also suggested that hyperactivity of this circuitry in response to negative versus positive emotional stimuli is attenuated following treatment with antidepressants.^4, 7, 12, 23, 24^ In a previous, placebo-controlled, parallel group study we found that that the amygdala hyperarousal exhibited by depressed patients in response to negative faces resolved after just 7 days of SSRI treatment and before significant changes in the clinical state.^13^ The latter study is important in showing that the effect of 7 days' SSRI treatment on neural responses in depressed patients cannot be attributed to the nonspecific effects of drug administration, repeat fMRI testing or changes in clinical state. The current investigation now provides support for the proposal that these early changes in neural processing of emotional information produced by antidepressant treatment are indeed important as a mechanism of later clinical response.

The amygdala, insula, ACC and thalamus are part of an integrated neural system, which is important for monitoring and affective response to emotional stimuli.^25^ Thalamic–amygdala connections have been proposed to be critical for the rapid monitoring and detection of salient stimuli for further processing.^26^ In depression, increased amygdala arousal has been suggested to create a bottom-up signal, outside of direct conscious awareness, that biases emotional stimulus processing across the higher cortical areas involved in a more complex processing; this results in maladaptive perceptions of the environment and social interactions.^2^ Indeed, a number of key studies in depression indicate that the hyperactivity of amygdala response exists even if the emotional valence of the stimulus is processed automatically by masking conscious perception.^2^ An early reduction in amygdala response to negative versus positive stimuli with SSRI treatment may therefore not be perceived as an immediate change in the subjective state. Instead, such a change in implicit processing would be hypothesised to set the scene for more adaptive perceptions of environmental stimuli, which then influence the conscious state over time.

Besides a decrease in amygdala responsiveness, the current study also highlights a role for early changes in the response of the cingulate and insula to emotional stimuli. These areas form key nodes in Mayberg's limbic–cortical model of depression^27^ and have been identified in meta-analyses of functional neuroimaging studies as underpinning negative biases of emotional processing in depression.^28, 29^ The current data set adds to this body of work, suggesting that emotion-specific modulation of response in this area following SSRI treatment may provide an early marker of therapeutic response.

It is of note that the difference in response to negative versus positive facial expressions largely seems to be driven by increased responses to happy cues rather than reduced responses to negative cues, although both effects may be present to some extent in areas like the amygdala. Similarly, differences between patients and controls at baseline in response to emotional cues seemed largely to be in response to the positive facial expressions. These observations are consistent with a growing focus on reduced positive processing in depression^30^ and also with behavioural evidence that increased perception of happy facial expressions rather than reduced processing of negative facial expressions is related to later clinical outcome with antidepressant drug treatment.^31^ It is difficult to draw definitive conclusions on a pattern extracted from this kind of fMRI analysis that reveals an overall interaction between positive versus negative cue processing; however, it suggests that a focus on the effects of boosting positive affective processing may be an important consideration in future research studies exploring early mechanisms of treatment response in depression.

In addition to the neural changes described above, 7 days' treatment with escitalopram was associated with some reduction in HAM-D scores, and this was numerically greater in later clinical responders. This raises the possibility that the change in neural activity found in the second scan in subsequent treatment responders might be secondary to this early improvement,^32^ rather than the neural changes leading to the subsequent clinical response. However, when this initial change in HAM-D score was accounted for in our statistical model, the early changes in neural response were still predictive of the therapeutic outcome. A secondary mediation analysis further suggested that early changes in neural response to emotional information mediate later clinical change, independent from any influence of early mood change. Therefore, early change in clinical symptoms does not seem to be driving the prediction provided by changes in emotional processing response.

Previous studies have identified differences in baseline (pretreatment) neural response to emotional material as a predictor of likely clinical response. In particular, increased perfusion and response in the rostral cingulate have been related to increased likelihood of response to antidepressant treatment.^33^ A recent PET study further suggested that basal perfusion of the insula may serve as a specific biomarker for response to drug versus psychological treatment,^34^ with increased perfusion related to a higher likelihood of remission with SSRI therapy, and the converse being true for cognitive behavioural therapy. However, the current pattern of results suggests that early change in neural response following SSRI treatment may be more sensitive to clinical response than baseline measures in fMRI. This approach has the potential to test the specific effects of a particular treatment at a given dose and time in patients at an early stage of the intervention.

Our findings have a number of clinical implications. First, they suggest that rapid change in emotional processing circuitry is related to clinical effectiveness of a particular treatment. As such, emotional processing models may usefully be applied in the drug-discovery programmes to facilitate decision-making about novel treatments in development. Critically, we have found early positive biases in emotional processing across antidepressant classes, including SSRIs, norepinephrine reuptake inhibitors and atypical antidepressants such as mirtazapine^10, 11, 35^ as well as treatments with novel mechanisms of action such as the N-methyl-D-aspartate receptor antagonist AZD6765.^36^ Second, as many patients do not respond to the first-line treatment with a given agent and waiting time for response can compound the delay in effective treatment, an early marker that could be used to tailor treatment in an individual would have significant advantages. It is also worth noting that relevant fMRI changes in emotional processing can be identified after single doses of antidepressant drugs in healthy volunteers.^8^ This raises the intriguing possibility that assessing the effect of single doses of medication in depressed patients might also have predictive value.

Future studies should, therefore, assess whether this early signature of antidepressant drug action can be used to predict response on an individual-patient level, using *a priori* defined neural and/or behavioural criteria and in a larger cohort of patients. It will also be necessary to examine whether these early changes in emotional processing predict placebo response, that is, function as a general marker of improvement rather than a specific drug-induced mechanism. Our study was limited by the absence of a placebo control condition to test this hypothesis, although in our earlier work we have found little influence of placebo treatment on emotional processing.^13^

In summary, this study provides the key evidence that, following the initiation of antidepressant treatment, early changes in the activity of neural systems responsible for emotional processing mediate later clinical response. These observations may be relevant to the development of improved therapeutic approaches to depression as well as for the early detection of clinical response.

## Figures and Tables

**Table 1:** Clinical and demographic data for week 6 responders (⩾50% decrease in HAM-D scores) versus non-responders (<50% decrease in HAM-D scores)

	*Responders*	*Non-responders*	*Responders* *versus* *non-responders*	*Healthy volunteers*	*Patients versus healthy controls*
Gender (F:M)	12:10	8:5	0.163, *P*=0.686	17:12	0.014, *P*=0.905
Age (mean (s.d.))	30 (11.5, 20–61)	30.1 (10, 20–52)	0.000, *P*=0.984	30 (9.6, 19–58)	0.004, *P*=0.949
Trait anxiety (mean (s.d., range))	59.4 (8.7, 43–74)	63.3 (10.3, 61–74)	1.427, *P*=0.421	29.6 (7.8, 20–44)	203.9, *P*<0.000
HAM-D (mean (s.d., range))	23.1 (4.9, 14–32)	22.9 (4, 14–29)	0.006, *P*=0.939	0.4 (0.8, 0–3)	707.4, *P*<0.000
BDI (mean (s.d., range))	31.6 (6.7, 19–49)	32.4 (5.3–40)	0.147, *P*=0.704	0.9 (1.5, 0–5)	691.5, *P*<0.000
Week 1 change in HAM-D (mean (s.d.))	–5 (6.8)	−1.9 (3.4)	2.373, *P*=0.133		
Week 6 reduction in trait anxiety	–13.4 (12.4)	−6.4 (10.8)	2.834, *P*=0.102		
Length of current episode (in months)	5.9 (6.0)	8.8 (8.7)	1.369, *P*=0.250		
Number of antidepressant-naive	8/22	6/13	0.326, *P*=0.568		

Abbreviations: ANOVA, analysis of variance; BDI, Beck Depression Inventory; HAM-D, Hamilton Depression Rating Scale; STAI, Spielberger's State-Trait Anxiety inventory.

Treatment resistance defined as a failure to respond to adequate courses (in terms of length and dose) of at least two antidepressants from different classes). Continuous variables are shown as mean (s.d.). There are no statistically significant differences between responders and non-responders. Gender and number of antidepressant-naive were performed using *χ*^2^-tests, all other comparisons suing ANOVA. Patients and controls differed in terms of clinical scores (BDI, HAM-D and STAI-T; all *P*=0.000), although there were no differences in terms of gender and age.

**Table 2:** Prediction of clinical response from an early change in neural response to fearful compared with happy facial expressions after 7 days' escitalopram treatment

	*MNI Coordinates, peak*			*Cluster size, voxels*	Z*-value*	P-*value*
	x	y	z			
Left amygdala/left insula	–40	–22	8	2204	3.76	2.15e^−09^
Anterior cingulate cortex	–8	4	38	2175	3.96	2.67e^−07^
Left supramarginal gyrus extending to left postcentral gyrus	–40	–28	32	876	3.66	0.0002
R supramarginal gyrus/right superior temporal gyrus	58	–32	2	712	3.56	0.0009
Bilateral thalamus	12	–22	10	601	3.18	0.003

Abbreviation: MNI, Montreal Neurological Institute.

Whole–brain analysis at *Z*=2.3 for responders versus non-responders.
