# Supplementary material for: Early changes in emotional processing as a marker of clinical response to SSRI treatment in depression
Source: Transl Psychiatry. 2016 Nov 22;6(11):e957–. doi: 10.1038/tp.2016.130 (PMC5314109; doi:10.1038/tp.2016.130)
Supplement: Supplementary Tables [file tp2016130x1.docx]

**Supplementary material**

**Supplementary Table 1** Greater neural response to fearful compared to happy faces across patients and control participants at baseline; whole–brain analysis at Z=2·3.

| Region | MNI Coordinates, Peak | | | Cluster Size, Voxels | Z value | P value |
| --- | --- | --- | --- | --- | --- | --- |
|  | x | y | z |  |  |  |
| R Insula/R Amygdala and Mid Temporal Gyrus | 564 | –14 | –6 | 771 | 3·96 | 0·001 |
| L Temporal Fusiform, Amygdala and Insula | –44 | -42 | -22 | 586 | 4.42 | 0·004 |
| L Angular gyrus/L Middle Temporal Gyrus | –40 | –54 | 20 | 569 | 3·88 | 0·005 |

**Supplementary Table 2** Prediction of clinical response from an early change in neural response to fearful compared to happy facial expressions after seven days’ escitalopram treatment, controlling for baseline depression HAMD, early change in HAM–D after one week of treatment, baseline trait anxiety and change in trait anxiety after 6 weeks of treatment; whole–brain analysis at Z=2·3.

| Region | MNI Coordinates, Peak | | | Cluster Size, Voxels | Z value | P value |
| --- | --- | --- | --- | --- | --- | --- |
|  | x | y | z |  |  |  |
| L Amygdala/L insula | –26 | –4 | –26 | 1566 | 3·78 | 1·79e^–07^ |
| Cingulate Gyrus (rostral, extending to anterior) | –4 | –26 | 26 | 787 | 3·66 | 0·0003 |
| R Superior Temporal Gyrus/Central Opercular Cortex | 52 | –22 | –8 | 577 | 3·78 | 0·003 |

**Supplementary Table 3** Prediction of clinical response from neural response to fearful compared to happy facial expressions after 7 days of treatment alone; whole–brain analysis at Z=2·3 for responders versus non–responders for 2 largest clusters and SVC for left amygdala.

| *Region* | *MNI Coordinates, Peak* | | | *Cluster Size, Voxels* | *Z value* | *P value* |
| --- | --- | --- | --- | --- | --- | --- |
|  | x | y | z |  |  |  |
| L Insula/Central Opercular Cortex extending to Anterior Cingulate Cortex and to R Insula; L Thalamus | 66 | –20 | 30 | 1489 | 4·06 | 1·19e^–26^ |
| Central Opercular Cortex/R Insula extending to R Frontal Pole | –28 | 56 | –27 | 7091 | 4·08 | 3·96e^–21^ |
| L Amygdala (SVC) | –24 | –2 | –26 | 18 | 2·9 | 0·038 |
